# *Campylobacter concisus* from chronic inflammatory bowel diseases stimulates IL-8 production in HT-29 cells

**DOI:** 10.1186/s13099-023-00532-5

**Published:** 2023-02-13

**Authors:** Marta Emilie Yde Aagaard, Karina Frahm Kirk, Hans Linde Nielsen, Rudi Steffensen, Henrik Nielsen

**Affiliations:** 1 Department of Infectious Diseases, Aalborg University Hospital, Hobrovej 18-22, 9000 Aalborg, Denmark; 2 Department of Clinical Medicine, Aalborg University, Søndre Skovvej 15, 9000 Aalborg, Denmark; 3 Department of Clinical Microbiology, Aalborg University Hospital, Mølleparkvej 10, 9000 Aalborg, Denmark; 4 Department of Clinical Immunology, Aalborg University Hospital, Urbansgade 32, 9000 Aalborg, Denmark

**Keywords:** *Campylobacter concisus*, Interleukin-8, Inflammatory bowel diseases, Microscopic colitis, Pathogenesis

## Abstract

The emerging pathogen *Campylobacter concisus* has been isolated from patients with gastrointestinal diseases; however, it is also present in the gut of healthy individuals. The aim of this study was to compare IL-8 production in HT-29 cells after infection with *C. concisus* from different gastrointestinal disease phenotypes. Additionally, to investigate whether differentiation of isolates in genomospecies (GS1 and GS2) or presence of the *zot* gene, encoding the Zot toxin, affects IL-8 production. A total of 37 *C. concisus* isolates from patients with microscopic colitis (n = 20), ulcerative colitis (n = 5), Crohn’s disease (n = 5), diarrhoea (n = 2) and from healthy controls (n = 5) were used. Intestinal HT*-*29 cells were infected and incubated for 24 h. Supernatants were subsequently removed and analysed for IL-8 by MILLIPLEX. All isolates were able to stimulate IL-8 production and IL-8 levels were higher than in non-infected HT-29 cells. No difference was observed between disease phenotypes or GS1 and GS2, whereas presence of the *zot* gene showed a tendency towards higher IL-8 production. Further investigations in other inflammatory and physiological models are needed to conclude whether *C. concisus* strains from different gastrointestinal disease phenotypes differ in pathogenic potential and play a part in gastrointestinal disease.

**Supplementary Information:**

The online version contains supplementary material available at 10.1186/s13099-023-00532-5.

## Introduction

*Campylobacter concisus* is an emerging *Campylobacter* species, prevalent in the human oral microbiota [1]. The bacterium is a Gram-negative, motile, curved or spiral-shaped rod capable of damaging epithelial barrier functions and invading intestinal cells in vitro [1, 2]. *C. concisus* is associated with prolonged diarrhoea [3, 4] and was recently found to be associated with microscopic colitis (MC), as *C. concisus* was demonstrated in saliva, faeces and mucosal biopsy samples from MC patients [5]. Furthermore, the risk of MC was also increased in patients with a previous *C. concisus* culture-positive stool sample compared with patients with culture-negative stool samples in a population-based cohort [6]. In contrast, the association with ulcerative colitis (UC) and Crohn’s disease (CD) is less clear [7]. The prevalence of *C. concisus* has been found to be significantly higher in mucosal biopsies from UC and CD patients than from healthy controls (HC) [1, 7]. However, similar risks of developing UC and CD in patients with a previous *C. concisus* culture-positive or culture-negative stool sample have been described [8], which suggest that the bacterium is a commensal with limited direct pathogenic effect on the intestinal mucosa. Nevertheless, *C. concisus* isolates have been found to be highly diverse in genetic studies [9, 10], and may therefore differ in pathogenic potential with respect to the site of isolation and disease phenotype. *C. concisus* can be divided into two genomospecies (GS1 and GS2) based on differences in the 23S rRNA gene [11]. GS2 isolates have been found to be more prevalent in mucosal biopsies compared to faeces and saliva, possibly reflecting differences in pathogenic potential between genomospecies [1, 10]. Furthermore, the putative virulence factor Zonula occludens toxin (Zot), which targets intestinal epithelial tight junctions, may increase the pathogenicity of the bacterium [1].

Previous studies have established that *C. concisus* is able to stimulate production of the pro-inflammatory cytokine IL-8 in intestinal cell lines and may therefore have pathogenic inflammatory effects on the intestinal mucosa [2, 12]. IL-8 is known to have an important but non-specific role in the pathogenesis of UC and CD [13, 14]. Interestingly, genetic cluster differentiation in *C. concisus* have affected expression of IL-8 mRNA in epithelial cells differently [15]; and Zot also has an effect on both IL-8 production and IL-8 gene expression[16, 17]. However, IL-8 production was similar in isolates from UC and CD patients and from HC, supporting no difference in the inflammatory activity in isolates from these gastrointestinal disease phenotypes [2, 12]. Nevertheless, most observations are from saliva and faecal isolates, while very few mucosal biopsy isolates have been studied, limiting the knowledge about effects of *C. concisus* isolated from the mucosal environment. Furthermore, the effect of MC isolates on IL-8 production has never been studied, since these isolates were only recently cultivated and as such, the pathogenic potential from this disease phenotype remains to be established.

The aim of this study was first to investigate the hypothesis that *C. concisus* mucosal biopsy isolates from different gastrointestinal disease phenotypes differ in pathogenicity by use of IL-8 production in HT-29 cells as a model for comparison; faecal, diarrhoeal isolates were also included in the study. Furthermore, we investigated whether GS distribution or prevalence of the *zot* gene, encoding Zot, has effect on IL-8 production.

## Methods

We used the human, adenocarcinoma, colonic cell line HT-29 [18] to compare the effect of different *C. concisus* isolates on IL-8 production in vitro*.* A total of 37 *C. concisus* isolates were used for infection. These isolates had previously been cultured from mucosal biopsy samples from MC patients with the two different subtypes collagenous colitis (CC) (n = 12) and lymphocytic colitis (LC) (n = 8) [5], as well as from patients with UC (n = 5) and CD (n = 5) and in addition from HC (n = 5) [7]. Furthermore, faecal isolates were used from patients with diarrhoea (D) (n = 2) [3]. *Campylobacter jejuni* ATCC 33560 was used as positive control. Additional file 1 shows data on the isolates used, including clinical information on patients and genomic data on GS distribution and presence of the *zot* gene, which was analysed by whole genome sequencing in previous studies [5, 10]. The Regional Ethics Committee of Northern Jutland approved the study (N-20160063).

HT-29 cells ACC 299 (DSMZ, Braunschweig, Germany) were maintained in Dulbecco’s Modified Eagle Medium (DMEM) with the supplementation of 10% foetal bovine serum (FBS) and 1% penicillin–streptomycin (10,000 U/ml) (Life Technologies, Carlsbad, USA), and incubated at 37 °C in humidified conditions with 5% CO_2_. Cell culture medium was replaced every 3–4 days, and cells were passaged after approximately 6–7 days. Cells were infected at passage 10–14. Forty-eight hours prior to infection, cells were trypsinised and seeded in 24-well plates with 1 × 10^5^ cells/cm^2^. Two hours before infection, cells were washed three times with PBS (Life Technologies, Carlsbad, USA) and thereafter incubated with antibiotic-free DMEM with 10% FBS.

All bacterial isolates were kept in sterile containers at -80 °C until seeded on 5% blood agar plates (SSI Diagnostica, Hillerød, Denmark) and incubated under microaerobic conditions (79.8% N_2_, 7.1% CO_2_, 7.1% H_2_, 6% O_2_) at 37 °C for 48 h prior to infection. Conditions were attained using the Anoxomat Mart II system (Mart Microbiology B.V., Drachten, Netherlands).

Prior to infection, isolates were liquefied in sterile, saline water to a McFarland value of 2.0 (approximately 6.0 × 10^8 CFU/ml) [19] using DensiCHECK™ Plus (BIOMÉRIEUX, Marcy l’Etoile, France) and HT-29 wells were promptly infected with a multiplicity of infection (MOI) of 200. The medium of negative-control wells was supplemented with an equivalent amount of sterile water. Cells were incubated for 24 h at 37 °C in humidified conditions with 5% CO_2_, and supernatants were subsequently removed and frozen in sterile containers at -80 °C until further analysis. Each experiment contained duplicate wells for each bacterial isolate and the negative control, and the experiment was repeated three times.

Supernatants were thawed, centrifuged for 10 min at 1000xg and analysed for IL-8 using the human cytokine/chemokine MILLIPLEX panel I kit (Cat. # HCYTMAG-60 K-PX41) (Merck Millipore, Billerica, USA). Assays were run as per manufacturer’s instructions with standards and samples in duplicates, overnight incubation at 4 °C (18 h, 750 rpm) and using a hand-held magnetic block for wash steps. Controls and standards were reconstituted in DMEM with 10% FBS. The basal amount of IL-8 production in HT-29 cells (negative wells) was subtracted from IL-8 production in *C. concisus* and *C. jejuni* wells*.*

Data were analysed using StataMP 16 (Statacorp LP, Texas, USA). All results are presented as a mean (SD). The Wilcoxon signed rank test was used for differences between two groups and Kruskal–Wallis one-way analysis of variance was used for groups of ≥ 3. A p-value < 0.05 was considered statistically significant.

## Results

All *C. concisus* isolates were able to stimulate IL-8 production in HT-29 cells. Mean production of IL-8 by each *C. concisus* isolate examined in this study is presented in the supplementary material. The basal IL-8 production in negative wells was 18.2 (2.7) pg/ml and IL-8 production by *C. jejuni* ATCC 33560 was 422.5 (89.4) pg/ml.

**Fig. 1:**
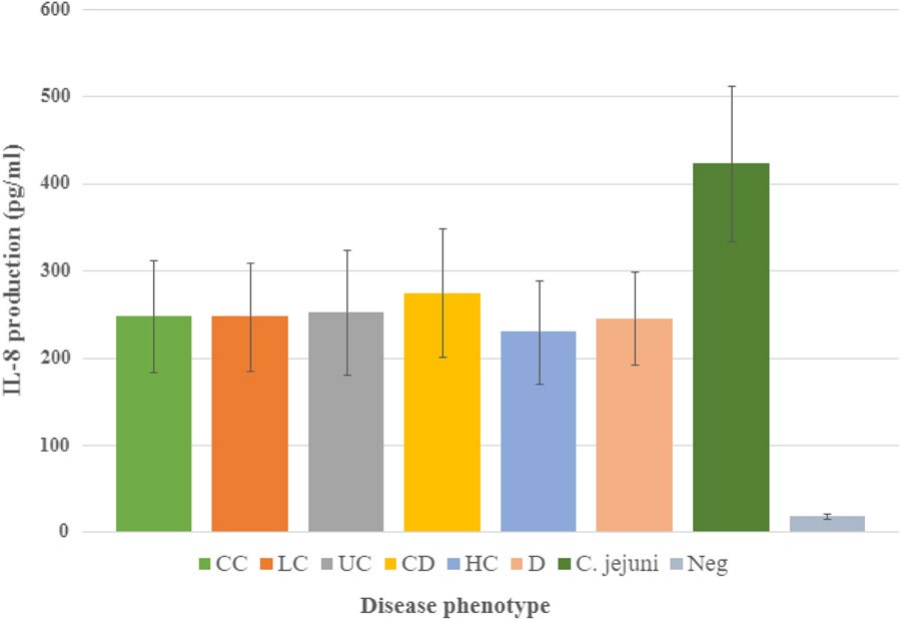
IL-8 production in HT-29 cells (multiplicity of infection of 200 for 24 h) by *Campylobacter concisus* isolates from different disease phenotypes. IL-8 production (pg/ml) is presented as mean (SD). The number of *C. concisus* isolates used were 12 CC (collagenous colitis) 248 (64.1); 8 LC (lymphocytic colitis) 247.3 (61.9); 5 UC (ulcerative colitis) 252.2 (71.2); 5 CD (Crohn's disease) 274.3 (73.6); 5 HC (healthy controls) 229.5 (59.1); 2 D (diarrhoea) 245.1 (53.2). *Campylobacter jejuni* (ATCC 33560) 422.5 (89.4) was used as positive control and uninfected cells as negative controls (Neg) 18.2 (2.7). Infection was performed in duplicate wells for each isolate used, and the experiment was repeated three times

When assessing IL-8 production based on gastrointestinal disease phenotype, we found no difference when comparing *C. concisus* isolates from CC 248 (64.1) pg/ml, LC 247.3 (61.9) pg/ml, UC 252.2 (71.2) pg/ml, CD 274.3 (73.6) pg/ml, HC 229.5 (59.1) pg/ml and D 245.1 (53.2) pg/ml, p = 0.3 (Fig. 1). Furthermore, IL-8 production did not differ between *C. concisus* isolates belonging to GS1 (n = 9) 242.5 (26.6) pg/ml and GS2 (n = 28) 252.4 (52.1) pg/ml, p = 0.79. There was no difference between IL-8 production in *C. concisus* isolates with or without the *zot* gene, whereas a tendency towards higher production was observed in *zot-*positive isolates (n = 15) 266.1 (55.5) pg/ml compared with *zot-*negative isolates (n = 22) 239 (37.8) pg/ml, p = 0.07.

## Discussion

This study set out to determine whether *C. concisus* mucosal biopsy isolates from different gastrointestinal disease phenotypes differ in pathogenicity, by use of IL-8 production in HT-29 cells as a model. The effects of biopsy isolates were further compared with two faecal isolates from diarrhoeic patients. We observed that all isolates stimulated IL-8 production in HT-29 cells. However, no differences were observed when comparing the IL-8 production based on gastrointestinal disease phenotype. This is in accordance with previous observations in HT-29 cells [2, 12]. Similar levels in IL-8 production were reported by Man et al. when comparing one biopsy isolate (CD) and two faecal isolates (one diarrhoeal and one healthy)[2]; and Ismail et al. found no significant difference when comparing strains from CD (saliva n = 4, biopsy n = 1) and UC (saliva n = 2, biopsy n = 1, luminal washout n = 1) patients and from HC (saliva n = 5) [12]. Finally, Kalischuk et al. observed no difference in IL-8 mRNA expression in T84 cells when comparing faecal isolates from diarrhoeic patients (n = 10) and from HC (n = 4) [15]. Based on these observations, the direct pathogenic importance of *C. concisus* in gastrointestinal inflammation can be questioned. Although wells infected with HC isolates produced the lowest amount of IL-8 in the present study, the difference was not statistically different from CC, LC, UC, CD and D isolates. Furthermore, *C. jejuni* produced higher levels of IL-8 than all *C. concisus* isolates; a control isolate chosen due to its well-known pathogenic effects in in vitro settings [20, 21]. Nevertheless, despite that *C. concisus* may be considered a commensal based on these observations, the bacterium still stimulates the innate immune system in a non-specific way and thereby hypothetically plays a part in mucosal inflammation if abundant in the gut microbiota. In addition, previous studies have shown that IL-8 is produced in colonic biopsies from both UC and CD patients and from HC; and that IL-8 levels correlate with higher grades of inflammation in UC and CD patients, whereas IL-8 levels in HC and uninflamed areas from UC and CD patients remain low [13, 22]. UC and CD patients appear to express equal levels of IL-8 in inflamed areas [13, 22]. IL-8 is considered an important chemoattractant of neutrophils and IL-8 levels also strongly correlate with neutrophil numbers in colonic tissue from UC and CD patients [22]. Interestingly, *C. concisus* was found to upregulate expression of the adhesion molecule CD11b on neutrophils exposed to the bacterium, and furthermore stimulate the oxidative burst response of neutrophils in a dose–response pattern [23]. However, the effects of isolates from HC have not previously been described [23]. The findings that *C. concisus* may be able to stimulate inflammatory response in the gut mucosa, both by stimulation of IL-8 production and activation of neutrophils should be further elucidated to conclude whether the bacterium has pathogenic, inflammatory potential in gastrointestinal disease.

To our knowledge, the present study is the first study to evaluate IL-8 production by *C. concisus* isolates from MC patients. MC is an inflammatory bowel disease with limited or no macroscopic changes in the colon; but microscopically with increased numbers of lymphocytes in the epithelium and lamina propria (CC and LC) and thickening of the sub-epithelial collagen band (CC) [24]. The pathogenesis of the disease is still sparsely described, but studies have reported increased amounts of cytokines related to a mixed Th17/Tc17 and Th1/Tc1 mucosal cytokine profile in mucosal biopsies [25, 26]. However, upregulated mRNA levels of these cytokines in CC and LC biopsies compared to HC biopsies have not corresponded with increased amounts of secreted proteins [25, 27], and Carrasco et al. furthermore found that the amount of IFN-ƴ (Th1) and IL-17-A (Th17) producing T-cells were lower in both CC and LC biopsies than in HC biopsies [27]. The cytokine profile of these disease entities therefore needs to be elucidated further to understand the inflammatory response in MC pathogenesis. Nevertheless, both IL-8 mRNA and protein levels are elevated in CC and LC biopsies compared to HC biopsies and this cytokine may therefore be part of inflammation in MC [28, 29].

We found no difference in IL-8 production between genomospecies in the present study. Likewise, Kalischuck et al. reported no difference in IL-8 mRNA expression when comparing infection with two separate genomospecies based on the 23S rRNA gene in T84 cells [15]. Observations from the present study support the hypothesis that GS differentiation based on 23S rRNA differences is not associated with the inflammatory potential of *C. concisus* [5, 10]. However, the number of GS1 strains used were low (n = 9) and the results should be interpreted with caution. Interestingly, prevalence of the *zot* gene showed a tendency towards higher IL-8 production. Zot was previously found to stimulate IL-8 production in HT-29 cells, with a markedly difference between IL-8 levels by purified Zot compared to an oral isolate of *C. concisus* with unknown *zot*-status [16]. The same pattern was observed when comparing the response in caco-2 cells to the *C. concisus* strain BAA-1457 (*zot*-positive) and purified Zot from BAA-1457 by RNA-sequencing [17]. Epithelial response was generally more specific to the Zot toxin but stronger when observing the strain; for instance, the toxin increased IL-8 gene expression, whereas BAA-1457 did not affect IL-8 gene expression but had effect on other inflammatory markers [17]. As the present study is based on few isolates with regard to *zot*-status it is difficult to conclude an effect of the *zot* gene on IL-8 production. Furthermore, some of our *zot*-negative isolates did stimulate higher levels of IL-8 than some *zot-*positive isolates. Whether Zot was expressed in the isolates used in the present study and vary in expression with regard to disease phenotype is unknown, as our data relies on genomic analysis. The toxin may have pathogenic, inflammatory potential but this possible association should be investigated by comparing purified toxins from different disease phenotypes in future studies.

The present study has some limitations. Firstly, to further describe the status of *C. concisus* as either a commensal or a possible pathogen, it would have been valuable to compare our results with the IL-8 production of other known non-pathogenic commensals of the gastrointestinal tract. Inclusion of a commensal such as *Escherichia coli* K12 as a control besides the known pathogen *C. jejuni* would strengthen future studies on the putative pathogenic role of *C. concisus.* Secondly, our model of comparing different strains focused on the production of IL-8 in HT-29 cells. Additional analysis of other cytokines or validation in other intestinal cell lines such as caco-2 or T84 cells may provide further comprehensive data on whether *C. concisus* isolates from different gastrointestinal disease phenotypes differ in inflammatory potential. We included mostly isolates from patients with MC, and statistical analysis comparing isolates from different disease phenotypes is therefore irrelevant. Thirdly, IL-8 production in intestinal cell lines cannot be directly correlated to the effects of the bacterium in vivo, where the effects of different strains most likely interact with other factors in the complex gut microbiota and mucosa. Laboratory settings such as freezing conditions and synthetic medias may also play a part in the pathogenic potential of the bacteria, as these may affect phenotypic expression of virulence factors. An animal model for testing *C. concisus* does not exist at the present time, but the inflammatory and physiological effects of the bacterium should be tested in such settings when possible. Finally, we did not know whether the Zot toxin was expressed phenotypically in our isolates and this information would have been valuable in analysing the effects of the toxin in intestinal inflammation.

In conclusion, all *C. concisus* isolates examined were able to stimulate IL-8 production in HT-29 cells. Although, IL-8 production did not differ between strains from different gastrointestinal disease phenotypes, the production was markedly higher than in HT-29 cells without bacterial infection. Furthermore, presence of the *zot* gene showed a tendency towards higher IL-8 production. *C. concisus* and possibly the Zot toxin may therefore play a part in intestinal inflammation and this association should be further elucidated in future studies.

## Supplementary Information

**Additional file 1: Table S1.** Characteristics and IL-8 production of the 37 *Campylobacter concisus *isolates used.

## Data Availability

The datasets generated and/or analysed during the current study are not publicly available yet but are available from the corresponding author on reasonable request.

## Abbreviations

IL-8: Interleukin-8
GS: Genomospecies
Zot: Zonula occludens toxin
MC: Microscopic colitis
UC: Ulcerative colitis
CD: Crohn’s disease
HC: Healthy controls
CC: Collagenous colitis
LC: Lymphocytic colitis
D: Diarrhoea
DMEM: Dulbecco’s Modified Eagle Medium
FBS: Foetal bovine serum
