# Supplementary material for: Campylobacter concisus from chronic inflammatory bowel diseases stimulates IL-8 production in HT-29 cells
Source: Gut Pathog. 2023 Feb 13;15:5. doi: 10.1186/s13099-023-00532-5 (PMC9926846; doi:10.1186/s13099-023-00532-5)
Supplement: Supplementary file 1 — Additional file 1: Table S1. Characteristics and IL-8 production of the 37 Campylobacter concisus isolates used. [file 13099_2023_532_MOESM1_ESM.docx]

**Additional file 1**

**Table 1: Characteristics and IL-8 production of the 37 *Campylobacter concisus* isolates used**

| **Patient ID** | **Disease phenotype*** | **Age (years)** | **Gender** | **Isolates** | **Genomo-species** | ***zot*** | **IL-8 production (pg/ml), mean (IQR)** |
| --- | --- | --- | --- | --- | --- | --- | --- |
| 5CC | CC | 75 | M | AAUH_5CCSigP1 | 2 | 0 | 179.9 (159.3-200.5) |
| 7CC | CC | 65 | F | AAUH_7CCSigD2@ | 1 | 0 | 229 (195.7-272.2) |
| 7CC | CC | 65 | F | AAUH_7CCSigD3 | 2 | 1 | 274.4 (249.5-299.3) |
| 8CC | CC | 88 | M | AAUH_8CCSigP1@ | 2 | 0 | 230 (198.3-257.3) |
| 13CC | CC | 72 | M | AAUH_13CCSigP1 | 2 | 0 | 218.9 (190.6-247.7) |
| 14CC | CC | 68 | M | AAUH_14CCDesc2 | 1 | 0 | 240.6 (217-274.9) |
| 19CC | CC | 68 | F | AAUH_19CCDesc1 | 1 | 0 | 221.3 (201.5-246.1) |
| 19CC | CC | 68 | F | AAUH_19CCDesc2@ | 2 | 1 | 435.4 (374.4-496.4) |
| 20CC | CC | 66 | M | AAUH_20CCSigD1 | 2 | 0 | 220 (196.8-234.7) |
| 27CC | CC | 65 | M | AAUH_27CCRec2 | 2 | 1 | 313 (299.6-323) |
| 29CC | CC | 70 | F | AAUH_29CCSigP2@ | 2 | 0 | 218.8 (191.8-240.1) |
| 31CC | CC | 72 | F | AAUH_31CCSigD2 | 2 | 0 | 242.6 (201.8-296.2) |
|  |  |  |  |  |  |  |  |
| 1LC | LC | 66 | M | AAUH_1LCDesc1 | 2 | 0 | 261.8 (198.5-354.7) |
| 1LC | LC | 66 | M | AAUH_1LCSigP1@ | 2 | 0 | 210.4 (182.4-257.1) |
| 6LC | LC | 56 | F | AAUH_6LCDesc2@ | 2 | 1 | 264 (210-327.4) |
| 6LC | LC | 56 | F | AAUH_6LCSigD2@ | 2 | 1 | 245.1 (190.2-321.7) |
| 6LC | LC | 56 | F | AAUH_6LCRec2@ | 2 | 1 | 243.4 (208.2-310.2) |
| 7LC | LC | 67 | F | AAUH_7LCDesc1@ | 2 | 1 | 205.4 (149.3-288.5) |
| 7LC | LC | 67 | F | AAUH_7LCSig1@ | 1 | 1 | 313.7 (274.2-364.1) |
| 7LC | LC | 67 | F | AAUH_7LCSig2@ | 2 | 1 | 234.7 (171.7-302.2) |
|  |  |  |  |  |  |  |  |
| 3UC | UC | 44 | M | AAUH_3UCce | 2 | 0 | 210.1 (195.3-237.5) |
| 9UC | UC | 45 | M | AAUH_9UCdp | 2 | 0 | 332.1 (279.6-375.4) |
| 35UC | UC | 23 | F | AAUH_35UCdp | 2 | 1 | 229.2 (166.7-297.5) |
| 43UC | UC | 67 | F | AAUH_43UCce@ | 2 | 1 | 258.4 (197.4-358.4) |
| 47UC | UC | 38 | F | AAUH_47UCil@ | 2 | 0 | 231 (179.9-326.6) |
|  |  |  |  |  |  |  |  |
| 12CD | CD | 63 | F | AAUH_12CDce | 1 | 0 | 259.2 (175.2-350.7) |
| 12CD | CD | 63 | F | AAUH_12CDtra@ | 2 | 0 | 297.8 (173.3-416.2) |
| 12CD | CD | 63 | F | AAUH_12CDrec@ | 2 | 1 | 275.6 (187-363.8) |
| 39CD | CD | 30 | M | AAUH_39CDti@ | 2 | 1 | 269.7 (218.4-308.7) |
| 39CD | CD | 30 | M | AAUH_39CDrec@ | 2 | 0 | 269.4 (214.8-341.1) |
|  |  |  |  |  |  |  |  |
| 3HC | HC | 44 | M | AAUH_3HCce | 2 | 0 | 305.9 (272.9-346.6) |
| 9HC | HC | 57 | M | AAUH_9HCce | 2 | 0 | 217.6 (211.1-224.2) |
| 9HC | HC | 57 | M | AAUH_9HCasc | 1 | 0 | 247.1 (238.7-260) |
| 14HC | HC | 69 | M | AAUH_14HCce | 1 | 1 | 199.8 (147.7-273.4) |
| 20HC | HC | 49 | F | AAUH_20HCrec@ | 2 | 0 | 173.1 (131-201.6) |
|  |  |  |  |  |  |  |  |
| 1D | D | 65 | M | 2010-376221 | 1 | 1 | 251.1 (172.2-312.5) |
| 2D | D | 57 | F | 2012-179281 | 1 | 0 | 239 (200.7-285.1) |

* CC (collagenous colitis), LC (lymphocytic colitis), UC (ulcerative colitis), CD (Crohn's disease), HC (healthy controls), D (diarrhoea)
